# Behaviors and influencing factors of Chinese oncology nurses towards frailty care: A cross-sectional study based on knowledge-attitude-practice theory in 2024

**DOI:** 10.1371/journal.pone.0313822

**Published:** 2025-01-09

**Authors:** Xiaoxue Chen, Fang Xiao, Yuhua Miao, Huiying Qin, Lirong Yang, Fang Shen, Xiuhong Yuan

**Affiliations:** Department of Gastric Surgery, State Key Laboratory of Oncology in South China, Guangdong Provincial Clinical Research Center for Cancer, Sun Yat-sen University Cancer Center, Guangzhou, P. R. China; National Trauma Research Institute, AUSTRALIA

## Abstract

**Background:**

The demand for frailty care is continuously increasing in hospitalized tumor patients with the aging of the population. Nurses are the primary care providers of hospitalized tumor patients with frailty but research on exploring their behavior and associated factors is limited. This study aims to describe the current situation of frailty care behaviors in oncology nurses and to explore the factors influencing frailty care behaviors.

**Methods:**

From August 1, 2023, to March 31, 2024, this cross-sectional study was conducted among nurses from oncology-related departments in 5 Secondary A (mid-tier hospitals providing comprehensive care) or above hospitals in South China. Convenience sampling was employed to recruit participants. In China, a "Secondary A hospital is a mid-tier hospital that provides comprehensive medical care and handles more complex cases than primary hospitals but is smaller and less specialized than tertiary hospitals. Data were collected using the standardized frailty-knowledge, attitudes, and practices questionnaire (F-KAP) and general information questionnaire including sociodemographic and work-related details. The sub-scale scores of knowledge, attitude, and practice were calculated by summing up the items within each sub-scale. Student’s independent t-test, one-way ANOVA, Pearson’s correlation coefficient, and two kinds of multiple linear regression models were used for data analysis.

**Results:**

We included a total of 17 (3.70%) male and 443 (96.30%) female participants in this study. The mean total score of oncology nurses for frailty care behaviors was 33.26±6.61. The three lowest scoring behaviors were “conduct frailty measurements and screening for patients (3.30±1.12)”, “accumulate frailty-related knowledge in daily work (3.59±0.87)”, and “actively pay attention to the patient’s debilitating condition”. Pearson’s correlations analysis showed that nursing grades (r = 0.13), frailty-related training willingness (r = 0.18), nursing frail patients experience (r = 0.22), frailty-related knowledge learning experience (r = 0.33), frailty-related training experience (r = 0.17), frailty care knowledge (r = 0.23), and hospice care attitudes (r = 0.54) were positively associated with frailty care behaviors. Two kinds of multiple linear regression models both showed that the factors most significantly associated with the oncology nurses’ frailty care behaviors are their self-rated subjective knowledge and attitudes towards frailty identification.

**Conclusion:**

Oncology nurses practiced relatively low-frequency frailty in daily work. Our findings provide theoretical support for improving frailty care attitudes among nurses and enhancing patient quality of life.

## Introduction

With ageing of the population, the incidence of tumors is increasing. In 2020, an estimated 19.3 million new cancer cases and nearly 10 million cancer-related deaths were reported globally, based on the GLOBOCAN 2020 data from the International Agency for Research on Cancer [1]; Furthermore, an increase of 47% in new cases of tumors by 2040 is projected [1]. According to global cancer statistics, among 185 countries, the incidence and mortality rates for 36 tumors in China rank in the moderate and above level. Tumors are a significant public health challenge that pose a threat to the physical and mental health of patients and impose a significant burden on healthcare systems as well as the economy [2]. For patients with tumors, who are affected not only by tumor invasion and metastasis but also various stressors such as having to undergo surgery, chemotherapy, radiotherapy and experience psychological distress, frailty is particularly common, with incidence rates up to 37.3% in China [3].

Frailty is defined as a multisystem clinical syndrome that involves a decrease in physiological reserve and increase in vulnerability to stressors; furthermore, in a frail state, the risk of dependency and death increases [4]. Although frailty is clinically characterized by age-related disturbances leading to unintentional weight loss, self-reported fatigue, generalized muscle weakness, and slow walking [5], several studies have demonstrated that patients of all ages with chronic health conditions or advanced disease may experience frailty symptoms [6, 7]. Frailty is an independent risk factor for unfavorable prognosis of patients with cancer and often triggers a series of health problems (e.g. increased risk of postoperative complications, toxic side effects of treatment, tumor progression, and death). Frailty also increases the demand for long-term care for patients with cancer, leading to a significant medical, financial, and psychosocial burden [8]. Thus, frailty in cancer patients during hospitalization is of research interest and warrants further study.

International Conference of Frailty and Sarcopenia Research International Clinical Practice Guidelines state that frailty care (FC) behaviors, including early identification and management of frailty, are potentially capable of reversing frailty [7]. Of note, nurses are not only the primary care providers for hospitalized patients with tumors but also the main implementers of FC [9]. Nevertheless, a few studies have found that among nurses in intensive care units (ICUs) and geriatric chronic disease-related departments, FC behaviors were not sufficiently implemented in daily practice and a suboptimal quality of FC was provided to patients [10–12]. In these studies, nursing practices were influenced by a diverse range of factors, including different ages of the nurses, types of departments, and tenure of nursing, and factors affecting nurses’ FC behaviors have not been definitively identified [10–12].

**Fig 1:**
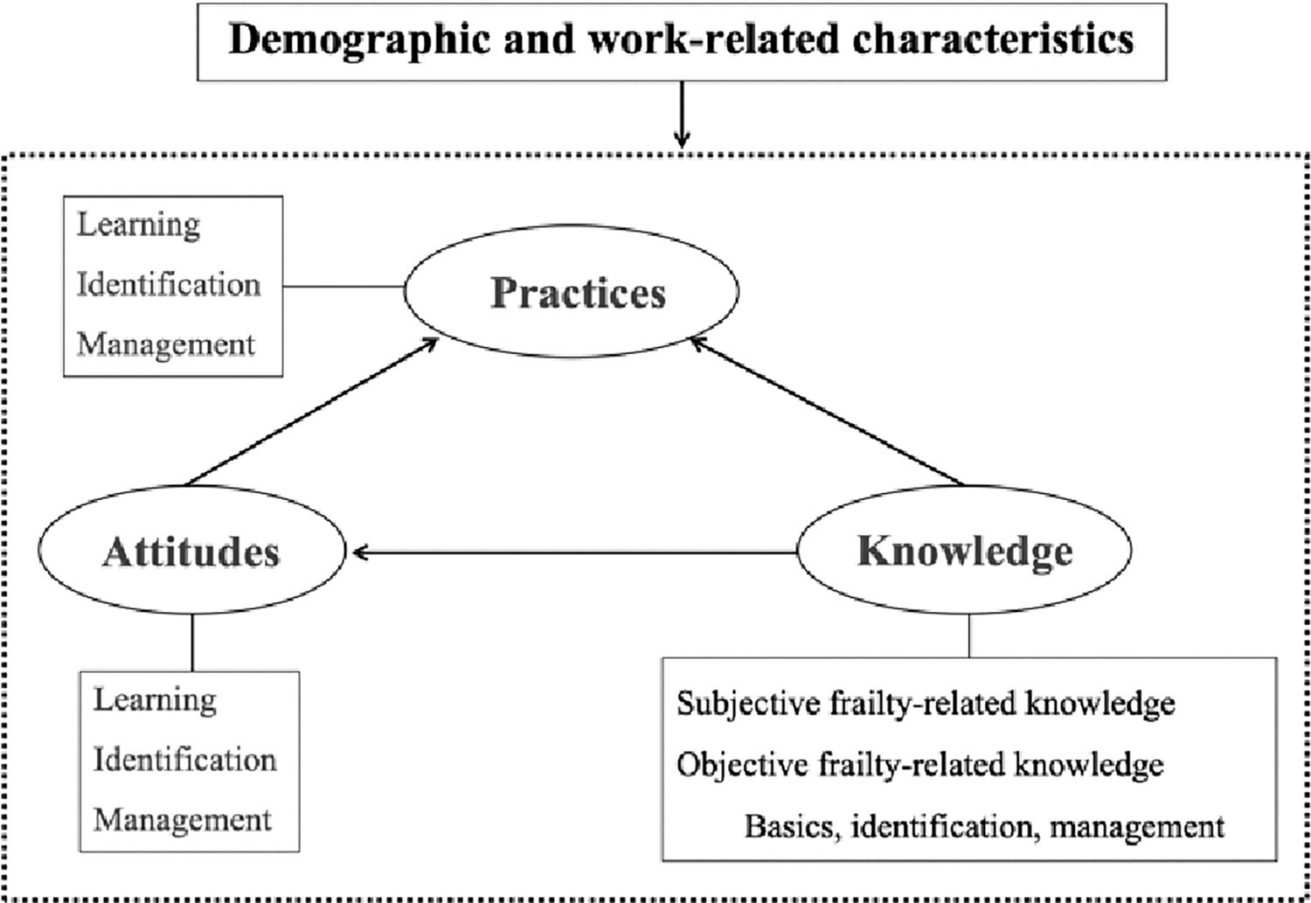
Conceptual framework.

Moreover, for the majority of patients with tumors, frailty does not occur or develop in ICUs or geriatric chronic disease-related departments. In fact, to our knowledge, research exploring the factors influencing FC behaviors of oncology nurses is still in its infancy, and no systematic studies have been conducted on FC behavior using any theoretical framework. The knowledge-attitude-practices (KAP) model, proposed by Mayo in the 1950s, is currently the most commonly applied in theoretical research on health-related behavior [13]. KAP holds that knowledge, attitudes, and behavior have a mutually and recursive complementary relationship in which knowledge acquisition and the formation of positive attitudes and beliefs influence human behavioral decision-making. As a well-established theory, KAP has been widely applied to explain health behavior among high-risk groups and predict how that behavior will change [14, 15]. KAP can therefore be used to analyze and interpret research on nurses’ FC behaviors. Fig 1 depicts the theoretical framework we hypothesized based on KAP in this study.

Accordingly, the objective of this study was to describe FC behaviors in oncology nurses and to apply KAP to explore the demographic factors, work-related factors, knowledge factors, and attitude factors that influence these behaviors. As well as providing a theoretical reference for supporting oncology nurses’ FC delivery, this study lays the foundation for improving the quality of life of frail patients with tumors.

## Materials and methods

### Objectives

This study aims to describe FC behaviors in oncology nurses and to explore, based on KAP, the demographic factors, work-related factors, knowledge factors, and attitude factors that influence these behaviors.

### Study design and setting

From August 1, 2023, to March 31, 2024, a cross-sectional study based on KAP was undertaken among nurses from oncology-related departments in 5 Secondary A or above hospitals in South China. In China, a "Secondary A hospital is a mid-tier hospital that provides comprehensive medical care and handles more complex cases than primary hospitals but is smaller and less specialized than tertiary hospitals. Ethical approval was granted by the Institutional Review Board of a cancer center (B2022-266-01). This study was performed in line with the Declaration of Helsinki. Brief information about this study was provided by the research team, and written informed consent through electronic signature was provided by participants prior to enrollment.

### Participants

The convenience sampling method was used to recruit all participants in this study. Inclusion criteria were as follows: (1) participants were aged 18 years or older; (2) they held a valid registered nurse license in China; (3) they were employed as full-time clinical frontline nurses, specifically those who directly provide clinical care to patients; (4) they had at least one year of experience working exclusively in oncology-related departments, including medical oncology, surgical oncology, radiation oncology, hematologic oncology, tumor biotherapy, and minimally invasive interventional oncology. No experience from non-oncology departments was included; and (5) they were willing to voluntarily participate in the study. Nurses were excluded if they were on holiday, on maternity leave, on sick leave, or out of the hospital on official duties.

### Measurements

Data were collected using a two-part questionnaire consisting of 1) questions seeking sociodemographic and work-related information; and 2) the Frailty-knowledge, attitudes, and practices (F-KAP) questionnaire aimed at patients with tumors–a standardized tool designed to assess oncology nurses’ frailty-related knowledge, attitudes, and behaviors.

### The general information questionnaire

A general information questionnaire was designed to meet study objectives and validated through testing by a panel of nurses from different hospitals. This questionnaire contained questions seeking regarding the demographic and work-related characteristics of participants, including their gender, age, marital status, educational level, hospital grade, department, nursing grade, advanced nursing ability, job duties, nursing tenure, tenure at oncology-related departments, nurse specialist or not, member of geriatric multidisciplinary teams or not, experience in nursing frail patients, frailty-related knowledge and learning experience, frailty-related training experience, willingness to receive frailty-related training, learning experiences, and learning expectations. Of these, hospital grade, nursing grade, and advanced nursing ability are categorized by the Management Criterion of the Ministry of Health in China. Furthermore, two open and multiple selection questions measured nurses’ learning experiences and expectations in regard to frailty-related knowledge. “How have you acquired knowledge in nursing frail patients?” addressed objective learning experiences through a choice of multiple responses, and “How do you wish to acquire knowledge of nursing frail patients?” was a subjective question to measure nurse’s learning expectations.

### Frailty-knowledge, attitudes, and practices questionnaire

**Fig 2:**
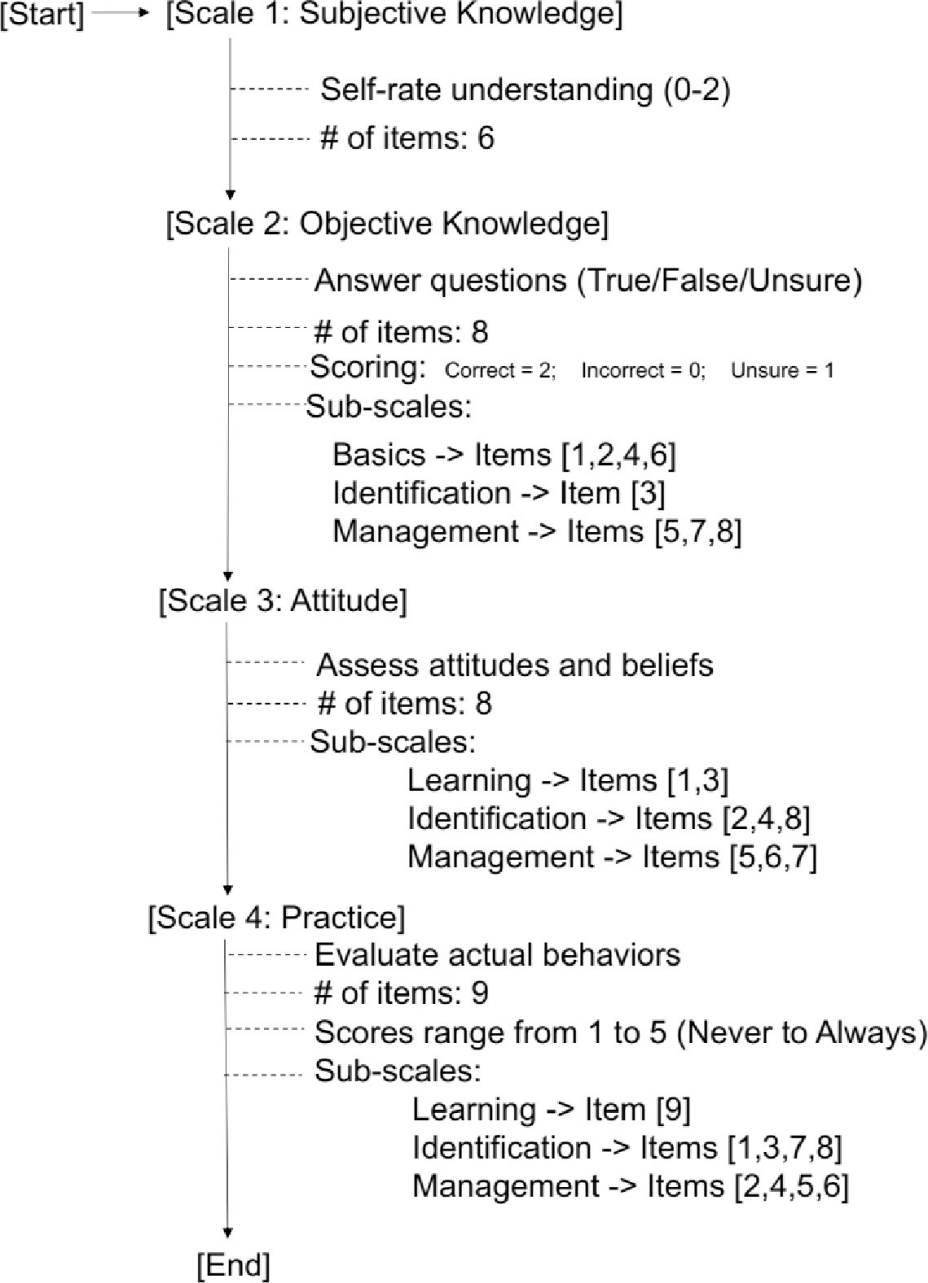
The flowchart of the frailty-knowledge, attitudes, and practices questionnaire.

Table 1 presents the structure of the F-KAP questionnaire regarding frail patients with tumors. The flowchart of the F-KAP questionnaire is presented in Fig 2. The questionnaire includes four scales. The first F-KAP scale asks participants to self-rate their understanding of frailty on a scale of 0 to 2—with higher scores indicating greater understanding—comprising 6 questions. This scale assessed subjective knowledge (self-rated). The second scale consists of 8 single-choice questions related to frailty with correct answers, with options being “true, false, unsure.” A correct answer is coded as 2, an incorrect answer is coded as 0, and choosing unsure is coded as 1. This scale assessed objective knowledge (ability). The third scale concerns participants’ attitudes and beliefs toward the FC of patients. The fourth scale seeks information on participants’ actual FC behaviors, with scores ranging from 1 to 5, corresponding to never, rarely, normal, often, and always, respectively. The various items from scales 2 to 4 can be further divided into three sub-scales according to their content: (1) learning of frailty knowledge (Learning), (2) identification of patient’s frailty (Identification), and (3) management of patient’s frailty (Management). The Cronbach’s α coefficient of the total scale was 0.909 (P<0.05) and the Cronbach’s α coefficient of each subscale ranged from 0.862 to 0.941 [11].

**Table 1:** The Structure of frailty-knowledge, attitudes, and practices questionnaire.

Part	Scale	# of items	Sub-scale	Items
1	Subjective knowledge (self-rated)	6	No	-
2	Objective knowledge (ability)	8	Basics	1, 2, 4, 6
			Identification	3
			Management	5, 7, 8
3	Attitude	8	Learning	1, 3
			Identification	2, 4, 8
			Management	5, 6, 7
4	Practice (behavior)	9	Learning	9
			Identification	1, 3, 7, 8
			Management	2, 4, 5, 6

To assess the frailty status of patients cared for by the interviewees during the study period, the Tilburg Frailty Indicator (TFI) scale was utilized. This frailty assessment tool, developed by Gobbens et al. at Tilburg University in the Netherlands, is based on an integrated frailty model [16]. The original scale was translated into Chinese by Xi et al. in 2013 [17]. The TFI scale comprises 15 items, categorized into three domains: physical (8 items), psychological (4 items), and social (3 items). The total score is the sum of the scores for each item, with a scoring range of 0 to 15 points. A score of 5 or above indicates the presence of frailty. The scale demonstrates strong consistency reliability, with a Kappa coefficient of 0.79 and stable internal consistency, as evidenced by a Cronbach’s alpha coefficient of 0.73. Furthermore, the Spearman correlation coefficients between the dimensions and the total score range from 0.422 to 0.889 (P < 0.01), indicating good construct validity.

### Survey method

The study utilized an online survey methodology via the Questionnaire Star platform (Changsha Ranxing Information Technology Co., Ltd., Changsha, P.R.C.) to recruit participants and collect data. Initially, the heads of nursing departments at each hospital were contacted and provided with a detailed explanation of the study’s objectives and significance. After receiving approval, the number of oncology nurses meeting the inclusion criteria was identified. The head nurses of oncology-related departments were then informed about the survey’s purpose, importance, and procedures to secure their cooperation and ensure smooth distribution and collection of the questionnaires.

A QR code containing a brief overview of the study, including background, significance, and methodology, was printed and posted on hospital bulletin boards. Participants were instructed to scan the QR code via WeChat to access the questionnaire and were assured that their responses would remain confidential and anonymous. Given the demanding nature of the nurses’ work, their shift patterns, and their higher level of education and professional expertise, the use of an online questionnaire was deemed appropriate for data collection. To ensure data quality and avoid duplicate responses, several measures were implemented on the platform: respondents could only submit the questionnaire once, the completion time had to exceed 10 minutes, and each mobile IP address was restricted to one submission. The platform also prevents participants from submitting uniform responses, ensuring their answers reflect actual circumstances. Once collected, responses were reviewed by two independent researchers who were not involved in data collection, and any questionnaires with illogical patterns or systematic response behaviors were excluded. Only valid responses were retained for further analysis.

### Sample size

A sample size of at least 5 to 10 times the number of items was required in the multiple linear regression analyses. In the F-KAP questionnaire regarding cancer patients with frailty, the number of items is 31 and the range of sample size was from 155 to 310. Given the possible potential loss (10%) of invalid samples or data, the minimum sample size required for this study was 171.

### Statistical analysis

Continuous variables were reported as mean ± standard deviation (SD) and compared using Student’s independent t-test or one-way ANOVA among groups. Categorical variables were presented as number and percentage. Pearson’s correlation coefficient was used to investigate the association between scales and sub-scales. Two kinds of multiple linear regression models were used to investigate the association between scales/sub-scales and FC behavior scores. The first method involved entering, in which all variables were entered into a model followed by comparison of the significant results and partial correlation; the second model used the forward method and Wald test to select the best combination of variables for predicting behavior results. All analyses were performed using IBM SPSS Version 27 (SPSS Statistics V27, IBM Corporation, Somers, New York). The statistical significance level for all the tests was set at P<0.05, two-tailed.

## Results

### Participants’ characteristics

A total of 17 (3.70%) male and 443 (96.30%) female participants were recruited. The largest age group was 25 to 35 years, comprising nearly two-thirds of respondents. One-third of the participants were single, two-thirds were married, and nearly 90% were college graduates. Almost all of the respondents (99.57%) were nurses at level 3, grade A hospitals; 1 from level 2 grade A and 1 from level 3 grade B. Table 2 contains other basic information about each participant.

**Table 2:** Participants’ characteristics (N = 460) and corresponding frailty care behavior results.

Parameters	N (%)	Behavior results (mean ± SD)	t/F	P
Gender			0.81	0.420
Male	17 (3.70%)	34.53 ± 7.45		
Female	443 (96.30%)	33.21 ± 6.58		
Age, year ^a^			0.04	0.963
<25	24 (5.22%)	33.58 ± 4.70		
25 to 35	296 (64.35%)	33.24 ± 6.82		
36 to 55	139 (30.22%)	33.18 ± 6.46		
Marriage status			0.12	0.886
Single	153 (33.26%)	33.24 ± 6.46		
Married	298 (64.78%)	33.23 ± 6.72		
Divorce	9 (1.96%)	34.33 ± 6.16		
Educational level			1.70	0.167
Technical Secondary School	3 (0.65%)	36.33 ± 8.50		
Junior College	22 (4.78%)	34.73 ± 7.09		
Bachelors	405 (88.04%)	33.32 ± 6.39		
Masters or above	30 (6.52%)	31.07 ± 8.61		
Department			1.62	0.168
Surgical Department	221 (48.04%)	33.08 ± 6.17		
Internal Medicine Department	194 (42.17%)	33.08 ± 6.99		
Outpatient Clinic	23 (5.00%)	34.22 ± 8.07		
Intensive care unit	11 (2.39%)	38.00 ± 5.44		
Emergency Department	11 (2.39%)	33.27 ± 5.00		
Nursing grade			4.02	0.008
Registered nurse	29 (6.30%)	29.72 ± 8.76		
Primary nurse	223 (48.48%)	33.95 ± 6.48		
Intermediate nurse	192 (41.74%)	32.89 ± 6.24		
Deputy senior or senior nurse	16 (3.48%)	34.44 ± 6.60		
Advanced nursing ability			1.50	0.190
N0	26 (5.65%)	31.58 ± 8.67		
N1	21 (4.57%)	31.48 ± 7.71		
N2	98 (21.30%)	32.71 ± 6.17		
N3	153 (33.26%)	33.17 ± 6.30		
N4	138 (30.00%)	34.03 ± 6.61		
N5	24 (5.22%)	35.00 ± 6.43		
Job duties			1.93	0.104
Responsible Nurse	346 (75.22%)	32.99 ± 6.52		
Nursing Team Leader	59 (12.83%)	34.32 ± 7.35		
Head Nurse	24 (5.22%)	35.13 ± 5.81		
Clinical teachers	23 (5.00%)	31.43 ± 5.77		
Others	8 (1.74%)	36.50 ± 7.50		
Nurse specialist			1.00	0.317
No	392 (85.22%)	33.13 ± 6.58		
Yes	68 (14.78%)	34.00 ± 6.80		

^a^ In the questionnaire, age was pre-set into four groups. Only one respondent was over the age of 55, and this respondent’s total behavior score was not included in the age group analysis.

Table 2 also demonstrated the mean scores of the practice scale according to the categories of each variable. The term ‘behavior result’ refers to the sum of scores from the nine items on the ‘practice’ sub-scale. As indicated, there were significant differences between participants with different nursing grades (P = 0.008), with registered nurses having relatively lower practice scores.

### The frailty condition of the patients

During the study period, a total of 144 patients were assessed using the TFI scale. The average score among the patients was 5.45 ± 3.07, with a median score of 5.5 (interquartile range: 3 to 7.5). Of these patients, 79 (54.86%) met the criteria for frailty.

### Participants’ nursing experience

Table 3 shows the nursing experience of participants, including the experience of frailty care. Three-quarters of the respondents have tenure of nursing ranging from 0 to 15 years, with the distribution of their nursing tenure being similar to that in the oncology department. About half of the respondents have experience in frailty care, but the majority have received related training and knowledge in frailty care. Most respondents indicated that they primarily acquire knowledge on frailty through channels such as departmental training, in-hospital training, colleague exchange, and reviewing guidelines and references. Very few engaged in external training or domestic and international learning exchanges. Over 80% of the respondents reported that they wish to acquire frailty-related knowledge through online courses, while nearly 60% of the respondents hoped to do so through offline training and training manuals.

**Table 3:** Participants’ nursing experience (N = 460) and corresponding frailty care behavior results.

Parameters	N (%)	Behavior results (mean ± SD)	t/F	P
Tenure of Nursing, years			1.09	0.360
≤5	106 (23.04%)	32.38 ± 6.61		
6 to 10	135 (29.35%)	33.32 ± 6.82		
11 to 15	104 (22.61%)	33.85 ± 5.86		
16 to 20	44 (9.57%)	32.50 ± 7.59		
>20	71 (15.43%)	34.07 ± 6.58		
Tenure at Oncology-related Department, years			0.83	0.510
≤5	121 (26.30%)	32.64 ± 6.58		
6 to 10	142 (30.87%)	33.11 ± 6.64		
11 to 15	98 (21.30%)	33.35 ± 6.65		
16 to 20	36 (7.83%)	33.58 ± 6.45		
>20	63 (13.70%)	34.46 ± 6.66		
Member of geriatric multidisciplinary teams			0.32	0.747
No	439 (95.43%)	33.24 ± 6.59		
Yes	21 (4.57%)	33.71 ± 7.11		
Experience in nursing frail patients			4.89	<0.001
No	237 (51.52%)	31.83 ± 6.75		
Yes	223 (48.48%)	34.78 ± 6.12		
Frailty-related knowledge learning experience			28.66	<0.001
No	212 (46.09%)	31.08 ± 6.95		
Yes, but not systematic	237 (51.52%)	34.86 ± 5.57		
Yes, and systematic	11 (2.39%)	40.73 ± 5.61		
Frailty-related training experience			3.71	<0.001
No	396 (86.09%)	32.81 ± 6.55		
Yes	64 (13.91%)	36.06 ± 6.36		
Frailty-related training willingness			3.88	<0.001
No	82 (17.83%)	30.73 ± 7.09		
Yes	378 (82.17%)	33.81 ± 6.38		
How have you acquired knowledge in nursing frail patients?			-^a^	-
Departmental training	186 (40.43%)	35.29 ± 6.04		
In-hospital training	167 (36.30%)	34.54 ± 6.38		
Colleague exchange	176 (38.26%)	35.30 ± 5.38		
Reviewing guideline and reference	148 (32.17%)	35.07 ± 6.58		
External training	42 (9.13%)	34.57 ± 7.42		
Domestic and international learning exchange	17 (3.70%)	34.65 ± 9.32		
Have not studied/learned before	165 (35.87%)	30.87 ± 7.12		
How do you wish to acquire knowledge of nursing frail patients?			-	-
Online course	385 (83.70%)	33.69 ± 6.50		
Offline training	271 (58.91%)	33.39 ± 6.52		
Training manual	270 (58.70%)	33.48 ± 6.98		
Others	3 (0.65%)	26.67 ± 2.08		

^a^ Not applicable.

Table 3 also demonstrated the mean scores of the practice scale according to the categories of each variable (behavior results). As indicated, those who had experience in nursing frail patients, frailty-related knowledge learning, or frailty-related training scored significantly higher on the practice scale compared with those who lacked such experience (all P<0.001). The participants who indicated their willingness to receive frailty-related training also scored significantly higher on the practice scale (P<0.001).

### Frailty-knowledge, attitudes, and practices questionnaire

Table 4 presents the average scores for the scales and sub-scales of the F-KAP questionnaire regarding patients with tumors, while Table 5 further displays the scoring results for all items on the practice scale as well as the execution rate (the proportion of respondents selecting ‘often/always’).

**Table 4:** Results from the Frailty-knowledge, attitudes, and practices scale.

Scale and sub-scale	Results (mean±SD)
Subjective knowledge (self-rated)	4.58 ± 2.93
Objective knowledge (ability)	13.78 ± 3.09
Basics	6.86 ± 1.64
Identification	1.50 ± 0.56
Management	5.41 ± 1.20
Attitude	32.53 ± 5.42
Learning	7.95 ± 1.50
Identification	12.04 ± 2.20
Management	12.54 ± 2.09
Practice	33.26 ± 6.61
Learning	3.59 ± 0.87
Identification	14.50 ± 3.11
Management	15.17 ± 3.00

**Table 5:** The results of the practice scale and items.

Items	Results (mean±SD)	Often/Always
1. Actively monitor patients for deterioration in their condition.	3.69 ± 0.88	277 (60.22%)
2. Communicate with patients about their limb muscle strength.	3.77 ± 0.86	306 (66.52%)
3. Conduct frailty measurements and screening for patients.	3.30 ± 1.12	212 (46.09%)
4. Promptly report patients ’ muscle strength condition to the doctors.	3.84 ± 0.85	318 (69.13%)
5. Provide effective early functional exercise guidance for elderly patients.	3.77 ± 0.89	300 (65.22%)
6. Guide family members to assist patients with appropriate activities to alleviate symptoms of physical weakness.	3.80 ± 0.84	308 (66.96%)
7. Promptly evaluate the nursing interventions for patients’ activities.	3.72 ± 0.90	296 (64.35%)
8. Promptly evaluate patients after implementing nutritional interventions.	3.78 ± 0.85	313 (68.04%)
9. Accumulate frailty-related knowledge in daily work.	3.59 ± 0.87	260 (56.52%)
Total practice score	33.26 ± 6.61	

### Correlation between scales

Table 6 presents the Pearson’s correlation coefficients among all scales and sub-scales. The results indicate that the respondents’ attitudes are most closely related to their FC behaviors, followed by their subjective knowledge, and, finally, their objective knowledge. Notably, all the coefficients in this table are statistically significant (all P<0.001). It was found that nursing grades (r = 0.13, P = 0.007), frailty-related training willingness (r = 0.18, P<0.001), nursing frail patients experience (r = 0.22, P<0.001), frailty-related knowledge learning experience (r = 0.33, P<0.001), frailty-related training experience (r = 0.17), P<0.001, frailty care knowledge (r = 0.23, P<0.001), and hospice care attitudes (r = 0.54, P<0.001) were positively associated with frailty care behaviors.

**Table 6:** Pearson’s correlation coefficient results among scales and sub-scales.

Pearson’s correlation coefficient	Subjective knowledge (self-rated)	Objective knowledge (ability)				Attitude				Practice			
		Basics	Identification	Management	All	Learning	Identification	Management	All	Learning	Identification	Management	All
Subjective knowledge (self-rated)	1												
Objective knowledge (ability)													
Basics	0.18	1											
Identification	0.19	0.60	1										
Management	0.14	0.85	0.48	1									
All	0.18	0.97	0.68	0.93	1								
Attitude													
Learning	0.21	0.31	0.22	0.30	0.32	1							
Identification	0.27	0.33	0.26	0.31	0.35	0.80	1						
Management	0.25	0.40	0.29	0.40	0.42	0.76	0.87	1					
All	0.26	0.37	0.28	0.36	0.39	0.89	0.96	0.95	1				
Practice													
Learning	0.37	0.20	0.19	0.14	0.20	0.42	0.45	0.41	0.45	1			
Identification	0.39	0.19	0.17	0.16	0.19	0.43	0.53	0.47	0.51	0.79	1		
Management	0.36	0.25	0.17	0.23	0.26	0.42	0.53	0.51	0.53	0.73	0.86	1	
All	0.40	0.23	0.18	0.20	0.23	0.45	0.55	0.51	0.54	0.83	0.97	0.96	1

The P-values for all coefficients are <0.01.

### Regression and partial correlation

Table 7 presents the results from both multiple linear regression models. As shown, the results are consistent regardless of whether all sub-scales are entered together in competition (entering method) or the best combination of variables is selected through an algorithm (forward method). The factors most significantly associated with the respondents’ practice scores are the respondents’ self-rated subjective knowledge and their attitudes towards frailty identification. These factors are not only significant but also exhibit the strongest association in terms of partial correlation coefficients, even after controlling for the influence of other sub-scales.

**Table 7:** Multiple linear regression results for frailty care behavior.

	Regression coefficient							Correlation	
Parameters	B	SE	95% CI lower	95% CI upper	β	t	P	r	Partial r
Entering method									
Subjective knowledge (self-rated)	0.60	0.09	0.43	0.78	0.27	6.86	<0.001	0.40	0.31
Objective Knowledge-basics	0.24	0.32	−0.38	0.87	0.06	0.76	0.445	0.23	0.04
Objective Knowledge-identification	−0.17	0.55	−1.26	0.91	−0.01	−0.32	0.752	0.18	−0.01
Objective Knowledge-management	−0.24	0.40	−1.03	0.54	−0.04	−0.61	0.544	0.20	−0.03
Attitude-learning	0.03	0.28	−0.52	0.58	0.01	0.12	0.907	0.45	0.01
Attitude-identification	1.13	0.25	0.63	1.62	0.37	4.44	<0.001	0.55	0.20
Attitude-management	0.34	0.26	−0.16	0.85	0.11	1.34	0.180	0.51	0.06
Forward method (Wald test)									
Attitude-identification	1.43	0.12	1.20	1.66	0.48	12.35	<0.001	0.55	0.50
Subjective knowledge (self-rated)	0.61	0.09	0.44	0.78	0.27	7.01	<0.001	0.40	0.31

## Discussion

The incidence of frailty among tumor patients has been reported to range between 37.3% and 64.7% in China, although the detection rate in hospitals is only 22.6% [3, 18]. Currently, very few hospitals or institutions perform frailty screening, assessments, and management as part of routine practice in China, and a unified standard for identifying and managing frailty has yet to be formed. Thus, the Asia-Pacific Clinical Practice Guidelines for the Management of Frailty recommended that the lack of participation in screening, assessing, and managing among healthcare providers should be urgently addressed [19].

In this study, we assessed the FC behaviors of 460 oncology nurses using the F-KAP questionnaire regarding patients with tumors and explored the demographic factors, work-related factors, knowledge factors, and attitude factors associated with these behaviors based on KAP. The mean score of nursing FC behaviors was 33.26 ± 6.61.

This study offers several contributions to the field of frailty care. Unlike global surveys such as Banna et al. [20], which included a broad range of healthcare professionals (n = 737 from 91 countries), this research focuses specifically on oncology nurses in China—a group that has been underrepresented and faces distinct challenges in frailty management. Using the KAP framework, this analysis moves beyond general awareness to examine how nurses’ knowledge and attitudes shape their care practices. This theoretical approach offers actionable strategies to improve frailty care at the nursing level. Moreover, the emphasis on the Chinese healthcare system, particularly in South China, highlights the need for locally tailored interventions. These insights fill a key gap in the literature, providing practical solutions not addressed by previous global studies.

Two kinds of multiple linear regression models using the entering, or forward method and the Wald test all showed that oncology nurses’ self-rated subjective knowledge and their attitudes towards frailty identification exhibited the strongest positive association with FC behaviors. The findings of this study indicated that oncology nurses practiced FC at a relatively low rate in daily work, consistent with previous studies. Several well-known frailty indices are commonly used in clinical practice., including the Fried Frailty Phenotype (FFP) [21], the Frailty Index (FI) [22], and the Clinical Frailty Scale (CFS) [23]. The FFP, a physical phenotype model, assesses frailty through five criteria: unintentional weight loss, exhaustion, weakness, slow walking speed, and low physical activity [21]. It is widely used due to its simplicity but focuses primarily on the physical components of frailty. In contrast, the FI [22], based on the accumulation of health deficits (e.g., symptoms, diseases, disabilities), offers a more comprehensive and multi-dimensional assessment, incorporating cognitive and psychological factors. The CFS [23], a nine-point scale based on clinical judgment, is frequently used in geriatric and acute care settings for a quick assessment of frailty severity. In comparison, our study utilized the F-KAP questionnaire, which emphasizes knowledge, attitudes, and practices (KAP) in frailty care, particularly in oncology nursing. While the aforementioned indices are patient-centered, the F-KAP is specifically designed to evaluate nurses’ competency in managing frailty, highlighting its relevance in the oncology setting. This underscores the need for specialized tools like the F-KAP in contexts where the complexity of frailty care demands not only physical assessments but also a deep understanding of care behaviors and attitudes."

The mean practice score of 569 nurses in geriatric departments in the study of Xuan et al. was 28.80 and that of 316 nurses in ICU in the study of Zhao et al. was 22.34 [11, 24]. The behavior of “conducting frailty measurements and screening for patients.” was the most inadequate, followed by “accumulating frailty-related knowledge in daily work” and “actively monitor patients for deterioration in their condition”, showing that frailty screening and assessments were frequently disregarded by oncology nurses. Our findings on frailty identification are consistent with those reported by previous studies [10, 12, 25, 26]. However, Warnier et al. showed that frailty screening forms part of nurses’ duties in two hospitals in the Netherlands, e.g., via pain assessments; therefore, these discrepancies in findings may have resulted from differences in study context, design, and setting [27].

The number of oncology nurses that implemented FC behaviors was small, which was possibly related to their nursing grades, willingness to receive frailty-related training, experience of nursing frail patients, frailty-related knowledge learning experience, and frailty-related training experience; these findings are in line with those of a previous study [28]. It is known that the professional experience and willingness to continue working of nurses in offering support and care to patients are major influencing factors in the quality of FC. We therefore proposed that in future research, adopt a more comprehensive measure is adopted to encourage nurses to accumulate more experience and promote a sense of calling to improve frailty identification and management.

Additionally, knowledge played a significant role in predicting FC behaviors, similar to the findings of Leblanc’s study. That study found that frailty-focused rater training improved accuracy of frailty assessment among 26 acute care nurses [29]. In the study of Warnier et al., nursing staff did not receive any specific training when the frailty screening tool was implemented, and it was suggested that nurses would benefit from a sort of traffic light (’alarm light’) that can provide frailty-related knowledge and identification guidance in the patient’s file [27]. Furthermore, the results of Hosie’s study emphasize that nurses’ recognition and assessment capabilities will be enhanced not only through continued professional development of the existing workforce but also through strengthened learning content in undergraduate nursing courses [30]. Thus, further research on nurse training, curriculum, and guidance is necessary to improve FC awareness among nurses.

Regarding individual attitude factors, this study revealed that nurses’ attitudes are closely associated with behavior; further analysis showed that their attitudes toward identifying frailty patients exhibited the strongest association in this context. These findings are in line with those of Dandan et al. and Zhao et al [27, 31], who studied the attitudes and behaviors of nursing staff in relation to hospice care and found that nurses’ positive attitudes toward hospice care led to better hospice care behaviors being implemented at an early stage. In addition, Coker et al. reported that nursing staff in the community emphasized the need for multi-domain frailty screening to support an interdisciplinary approach to care of older patients [32]. Warnier et al. reported that nurses emphasized the importance of tools for frailty screening. However, these tools as well as their summative scores were not promptly used during regular working routines [27]. Further discussion to explain the inconsistency between attitude and FC behavior in regard to frailty identification would be worthwhile.

### Limitations

Several limitations should be taken into account when interpreting our results. The first limitation is the cross-sectional design of this study, which focused on correlation between FC behaviors and influencing factors rather than causality. Hence, the conclusions on causal effects among the variables should be tested in more detail in subsequent longitudinal studies. A further limitation of this study lies in the convenience sampling method, which may result in selection bias and limit extrapolation of the results. Future studies should target more hospitals of different areas and grades as well as make dedicated efforts to use better sampling methods and larger sample sizes to enhance reliability and generalizability of the findings. The third limitation is that this study did not consider certain potential influencing factors such as tumor type, tumor grade, psychological conditions, nursing staffing, and nursing salary package. Accordingly, future studies exploring additional factors should be carried out to provide a more comprehensive analysis of nurses’ FC behaviors. Lastly, this study did not assess other potential barriers to practicing frailty assessment, such as access to resources and time constraints. These factors may significantly impact nurses’ ability to conduct frailty assessments in actual clinical settings.

## Conclusion

The FC behaviors of oncology nurses in 5 Secondary A or above hospitals in South China, and the factors influencing these behaviors, were analyzed in this study. The research findings showed that oncology nurses practiced FC at a relatively low frequency in their daily work, and more interventional measures should be adopted to promote better FC behaviors toward frail patients with tumors. Our findings also suggest several directions, including acquisition of frailty knowledge and enhancement of attitudes towards frailty, for future exploration and the development of possible intervention to promote FC behaviors among oncology nurses. In addition to providing theoretical support for enhancing FC behavior among nurses, this study also establishes the foundation for improving the quality of life of frail patients with tumors.

## Supporting information

S1 File Dataset. Dataset containing information used in the study analysis, for each participant. Original data, in Chinese, was translated to English. Data was shared in accordance with participant consent and approved by the Institutional Review Board of Sun Yat-sen University Cancer Center.
(XLSX)
